# Clinical feasibility of NGS liquid biopsy analysis in NSCLC patients

**DOI:** 10.1371/journal.pone.0226853

**Published:** 2019-12-20

**Authors:** Eirini Papadopoulou, Nikolaos Tsoulos, Katerina Tsantikidi, Vasiliki Metaxa-Mariatou, Pinelopi Eleftheria Stamou, Athina Kladi-Skandali, Evgenia Kapeni, Georgios Tsaousis, George Pentheroudakis, Dimitrios Petrakis, Dimitra Ioanna Lampropoulou, Gerasimos Aravantinos, Ioannis Varthalitis, George Kesisis, Ioannis Boukovinas, Pavlos Papakotoulas, Nikolaos Katirtzoglou, Elias Athanasiadis, Flora Stavridi, Christos Christodoulou, Anna Koumarianou, Yeşim Eralp, George Nasioulas

**Affiliations:** 1 GeneKor MSA, Athens, Greece; 2 Department of Medical Oncology, School of Medicine, Ioannina, Greece; 3 Society for Study of Clonal Heterogeneity of Neoplasia (EMEKEN), Ioannina, Greece; 4 Second Department of Medical Oncology, Agii Anargiri Cancer Hospital, Athens, Greece; 5 1st Oncology Department Henry Dunant Hospital Center, Athens, Greece; 6 Oncology Department, Saint Luke Private Hospital, Thessaloniki, Greece; 7 BioClinic Thessaloniki, Thessaloniki, Greece; 8 First Department of Clinical Oncology, Theagenio Hospital, Thessaloniki, Greece; 9 Euroclinic, Athens, Greece; 10 Department of Medical Oncology, Mitera Hospital, Athens, Greece; 11 Fourth Department of Medical Oncology, Hygeia Hospital, Athens, Greece; 12 Second Department of Medical Oncology, Metropolitan Hospital, Athens, Greece; 13 Hematology Oncology Unit, Fourth Department of Internal Medicine, Attikon University Hospital, Athens, Greece; 14 Department of Medical Oncology, Istanbul University School of Medicine, İstanbul, Turkey; Virginia Commonwealth University, UNITED STATES

## Abstract

**Background:**

Analysis of circulating tumor nucleic acids in plasma of Non-Small Cell Lung Cancer (NSCLC) patients is the most widespread and documented form of "liquid biopsy" and provides real-time information on the molecular profile of the tumor without an invasive tissue biopsy.

**Methods:**

Liquid biopsy analysis was requested by the referral physician in 121 NSCLC patients at diagnosis and was performed using a sensitive Next Generation Sequencing assay. Additionally, a comparative analysis of NSCLC patients at relapse following EGFR Tyrosine Kinase Inhibitor (TKIs) treatment was performed in 50 patients by both the cobas and NGS platforms.

**Results:**

At least one mutation was identified in almost 49% of the cases by the NGS approach in NSCLC patients analyzed at diagnosis. In 36 cases with paired tissue available a high concordance of 86.11% was observed for clinically relevant mutations, with a Positive Predictive Value (PPV) of 88.89%. Furthermore, a concordance rate of 82% between cobas and the NGS approach for the *EGFR* sensitizing mutations (in exons 18, 19, 21) was observed in patients with acquired resistance to EGFR TKIs, while this concordance was 94% for the p.T790M mutation, with NGS being able to detect this mutation in three 3 additional patients.

**Conclusions:**

This study indicates the feasibility of circulating tumor nucleic acids (ctNA) analysis as a tumor biopsy surrogate in clinical practice for NSCLC personalized treatment decision making. The use of new sensitive NGS techniques can reliably detect tumor-derived mutations in liquid biopsy and provide clinically relevant information both before and after targeted treatment in patients with NSCLC. Thus, it could aid physicians in treatment decision making in clinical practice.

## Introduction

Non-Small Cell Lung Cancer (NSCLC) is one of the most common and aggressive tumor types, nevertheless, it is also the tumor type with the majority of approved targeted agents available. [1, 2]. Analysis of tumor molecular profile can provide predictive information to guide treatment in these patients. Due to frequent low availability of tissue, simultaneous analysis of targetable mutations using multigene tests seems to be the most appropriate approach, saving both time and tissue availability. Next-generation sequencing (NGS) analysis is a robust technology that has been widely used for the detection of aberrations in genes that can be used as biomarkers of response to treatment, such as *EGFR*, *ALK*, *ROS1*, *BRAF*, *NTRK1*,*2 & 3*, *ERBB2*, *RET* and *MET* [3, 4].

For decades the only available material for molecular profiling was considered the patient’s Formalin Fixed Paraffin Embedded (FFPE) tumor tissue. The FFPE material has several advantages since it is a widely available material, easy to use and store [5, 6]. In addition, it provides the possibility of selecting the most appropriate cancer tissue either by microdissection or by macrodissection, increasing the sensitivity of somatic mutation detection assays [7, 8]. However, FFPE material also has several disadvantages, such as its unavailability in cases of inoperable tumors and its inability in some cases to capture the tumor’s heterogeneity [9]. Moreover, the genetic material obtained due to paraffin processing of tissue, is sometimes of poor quality and not adequate for molecular analysis [5, 10]. Most importantly, the tumor’s molecular profile is altered, mainly following targeted therapy and those alterations cannot be detected by analyzing the primary tumor material [11–13] but require invasive tissue re-biopsies.

The presence of neoplastic characteristics in the plasma DNA of cancer patients was first reported back in 1989 [14]. In the following years, several studies have shown that the analysis of cell-free tumor-derived nucleic acids in cancer patient’s body fluids (plasma, serum, Bronchoalveolar lavage, urine, stool, etc.) can be used to detect tumor specific alterations [15]. The term Liquid Biopsy has emerged indicating the use of these non- or minimally invasive materials for tumor characterization. The mutation status detected in a liquid biopsy reflects the status present in the patient’s tumor. Additionally, circulating tumor nucleic acids (ctNA) analysis could eventually detect more somatic alterations compared to the analysis of a specific area in a FFPE tissue, since it originates from the whole tumor’s area and/or metastasis present in the patient’s body, thus being more representative of intra and inter-tumor heterogeneity [16–18].

The use of plasma samples for ctNA analysis has nowadays become feasible due to the development of sensitive molecular techniques that can detect with high accuracy minimal amounts of ctNAs that are present in this material. For this purpose a variety of methods have been used, including digital PCR, Real-time PCR, Arms PCR, BEAMing technology and Next Generation Sequencing (NGS) [19–21].

Currently, the only FDA (Food and Drug Administration) approved test for *EGFR* mutation detection in plasma is the cobas® EGFR Mutation Test v2. Even though this approach meets the prerequisites for reliable detection of tumor-specific mutations in plasma, it has the disadvantage of being a single gene test analyzing specific mutations [20, 22–24]. On the other hand, NGS is the only methodology that can be used for the simultaneous analysis of multiple genes or even the entire cancer genome. This is of great importance in the era of individualized treatment, where the number of genes and gene alterations, targeted by drugs is increasing continuously, especially in tumors with many targetable gene alterations present such as NSCLC. Thus, NGS can be used to provide a broad molecular profile either in tissue or in plasma. Since it is the only method that provides a comprehensive analysis of many biomarkers simultaneously, it is becoming a tool of great clinical utility. The importance of such an approach is more prominent for tumors such as NSCLC, with limited tissue available but with an abundance of biomarkers that could guide treatment decisions. The simultaneous analysis of all tumor variations can be cost and time saving without the waste of valuable tissue material. Thus, liquid biopsy analysis can be used for tumor monitoring and for early detection of molecular relapse. Furthermore, the presence of resistance mutations due to treatment can be identified and eventually lead to the modification of the treatment plan in these patients [3, 25].

The most prevalent resistance mutation in this setting is the T790M in exon 20 of the *EGFR* gene, which can be targeted by third-generation TKIs. However, several other gene alterations are also implicated in Tyrosine Kinase Inhibitors (TKIs) resistance and NGS is the most appropriate method for the analysis of these resistance mechanisms. Moreover, resistance mutations that arise following treatment with third-generation TKIs (such as the C797S mutation in the *EGFR* gene) can also be detected by this methodology [11, 26]. Plasma ctNA NGS analysis has been shown to be a reliable alternative to tissue biopsy analysis and its use in clinical practice is constantly increasing [27, 28].

The aim of this study was to investigate the feasibility of ctNA analysis in everyday practice, using a sensitive NGS approach in patients with NSCLC. The plasma mutation distribution in newly diagnosed patients was calculated. Furthermore, a comparison among the results obtained by cobas and NGS was carried out in patients at progression on treatment with *EGFR* TKIs.

## Material and methods

### Patient selection

**Fig 1:**
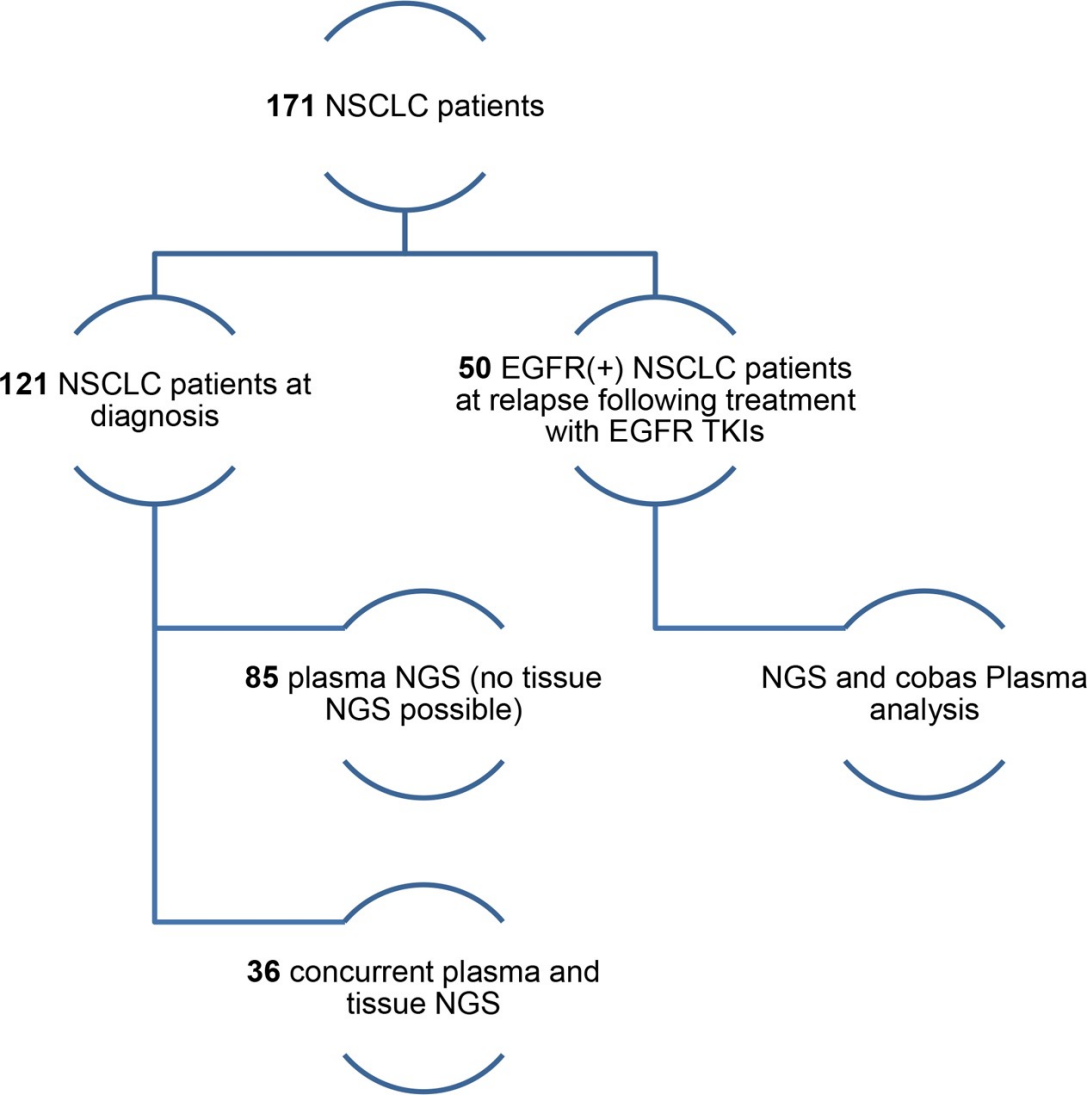
Study design.

In the current study, plasma liquid biopsy analysis was conducted using the NGS and/or cobas methods in 171 NSCLC patients who were referred to our laboratory between January 2017 and June 2019. Of those 121 were newly diagnosed without a previous treatment assigned. In the majority of the cases (85), a liquid biopsy analysis was requested due to the unavailability of a tissue sample, while in 36 patients both tissue and liquid biopsy were available. Additionally, 50 patients that were positive for an *EGFR* sensitizing mutation at diagnosis and had received targeted treatment were tested by both the cobas *EGFR* mutation test and NGS, at relapse (Fig 1).

The study was approved by the ethical committee of “Agii Anargiri” Cancer Hospital. All patients gave informed consent for molecular analysis in blood and tissue, in accordance with the Declaration of Helsinki.

### Cell-free Total Nucleic Acids (cfTNA) isolation

10 ml of blood samples were collected in Cell-Free DNA BCT tubes (Streck, La Vista, NE), that are used to stabilize Cell free Total Nucleic Acids (cfTNA). Plasma was separated from the cellular fraction by centrifugation twice at 1800 rcf for 10 min at 4 ^o^C. cfTNA was isolated from 2 ml of plasma using QIAamp Circulating Nucleic Acid Kit (Qiagen) according to the manufacturer’s instructions. cfTNAs’ concentration was measured with the use of the Qubit^™^ 2.0 Fluorometer in combination with the Qubit^™^ ssDNA Assay Kit (Thermo Fisher Scientific).

### Tissue selection and DNA extraction

Hematoxylin and eosin stained sections of formalin-fixed and paraffin-embedded (FFPE) tumor biopsies from all samples were reviewed to ensure a tumor cell content of >75%, where possible and the tumor area was marked by a pathologist. Genomic DNA was extracted from unstained 10 μm thick FFPE sections using the QIAmp FFPE tissue kit (Qiagen). The DNA concentration of all samples was determined spectrophotometrically (NanoDrop2000, Thermo Fisher Scientific). FFPE extracted DNA concentration was measured using the Qubit^™^ 2.0 Fluorometer in combination with the Qubit^™^ dsDNA HS assay kit (Thermo Fischer Scientific).

### Next generation sequencing libraries preparation

The NGS for FFPE DNA analysis was conducted using a custom Ion AmpliSeq panel which was based on the Ion Ampliseq Colon and Lung Cancer Research Panel v2 with an additional amplicon in the *MET* gene (to include the exon 14 skipping mutations) and two amplicons of exons 2 and 3 of the *HRAS* gene [29]. Fusion RNA transcript analysis was performed using the Ion AmpliSeq^™^ RNA Fusion Lung Cancer Research Panel (Thermo Fisher Scientific). Details of the target regions of the 23-gene panel are available upon request. The genes analyzed include *AKT1*, *ALK*, *BRAF*, *CTNNB1*, *DDR2*, *EGFR*, *ERBB2*, *ERBB4*, *FBXW7*, *FGFR1*, *FGFR2*, *FGFR3*, *KRAS*, *MAP2K1*, *MET*, *NOTCH*, *NRAS*, *PIK3CA*, *PTEN*, *SMAD4*, STK11, *TP53*, *HRAS*. An amplicon library was generated from 10ng of total FFPE extracted DNA or 6 μl of ctDNA, using the Ion AmpliSeq Library Kit 2.0 (Thermo Fischer Scientific) according to the manufacturer’s instructions. Briefly, amplicon amplification was performed using Ion Ampliseq^™^ HiFi Master Mix (Thermo Fischer Scientific). The amplicons were then digested with FUPA reagent and barcoded with the IonCode^™^ Barcode Adapters 1–384 Kit (Thermo Fischer Scientific). Subsequently, the amplified products were purified from the other reaction components using Agencourt AMPure XP PCR purification system (Beckman Coulter).

The NGS for cfTNA analysis was conducted using Oncomine Lung Cell-Free Total Nucleic Acid Research Assay (Thermo Fisher Scientific). The assay uses random molecular tags that are used as unique molecular identifiers (UMI) to uniquely label each molecule prior to library amplification. In this way, randomly incorporated errors can be distinguished (and removed) from true variants, increasing the method’s accuracy and minimizing false positives. The assay includes the analysis of Hotspot genes (Single Nucleotide Variations and short insertions/deletions): *ALK*, *BRAF*, *EGFR*, *ERBB2 (HER2)*, *KRAS*, *MAP2K1*, *MET*, *NRAS*, *PIK3CA*, *ROS1*, and *TP53*. Furthermore, the assay includes the analysis of RNA fusions that involve 3 fusion driver genes: *ALK*, *RET*, *ROS1*. Copy number variations are also calculated for the following genes: *MET* and *ERBB2*. cfTNA concentration was measured with the Qubit^™^ 2.0 Fluorometer using the Qubit^™^ ssDNA Assay Kit (Thermo Fischer Scientific). Library preparation was performed according to the manufacturer’s provided protocol. Briefly, Reverse Transcription of the cfTNA was carried out using SuperScript^™^ VILO^™^ Master Mix. The Cell-free total nucleic acid reverse transcription products were amplified with a 2 cycle PCR to copy each strand of an original DNA fragment into a fragment with random sequences (Tags) and A/P1 adaptors attached to 5’/3’ ends. Following that, a bead purification step was performed using Agencourt AMPure XP PCR purification system (Beckman Coulter, Inc., Brea, CA, USA). Subsequently, an 18 cycle amplification of the tagged amplicons was performed with a unique barcode for each sample, using the Tag Sequencing Barcode Set (Thermo Fischer Scientific). Purification and size selection of the barcoded library was achieved using Agencourt AMPure XP PCR purification system. Finally, the concentration of each library was determined by real-time PCR, using the Ion Library TaqMan® Quantitation Kit (Thermo Fischer Scientific)

Successively, 100pmoles of the DNA or ctTNA libraries were separately combined and clonally amplified on Ion sphere^™^ particles (ISP) by emulsion PCR performed on the Ion One Touch^™^ 2 instrument with the Ion 540^™^ Kit-OT2 (Thermo Fisher Scientific) according to the manufacturer’s instructions.

Quality control was performed using the Ion Sphere^™^ Quality Control kit (Thermo Fisher Scientific) to ensure that 10–30% of template-positive ISP were generated in the emulsion PCR. Finally, the template-positive Ion Sphere^™^ particles were enriched using the Ion OneTouch^™^ ES instrument, loaded on an Ion 540^™^ Chip and sequenced on an Ion GeneStudio^™^ S5 Prime System Sequencer according to the manufacturer’s instructions. All samples were processed in duplicate. In case of a discordant result a new duplicate NGS experiment was performed. A variant was considered as true when it was detected in at least 3 out of the 4 NGS runs.

### NGS data analysis

NGS data analysis was performed with the Ion Reporter^™^ 5.10.1.0 software directly from within Torrent Suite^™^ 5.10.1 software (Thermo Fisher Scientific), followed by manual inspection, along with the commercial analysis software Sequence Pilot version 4.3.0 (JSI medical systems, Ettenheim, Germany). The coverage analysis was performed using the coverage analysis plug-in v5.0.4.0. The statistics generated from this plugin were used to evaluate the quality of each library in the sequencing run. For FFPE DNA libraries, the copy number variation (CNV) analysis was performed using the Ion Reporter^™^ Software. CNVs were reported based on their copy number relative to the control sample used. The software reports all possible CNVs assigning a score, with scores >10 indicating high-confidence CNVs. This value was used as a threshold for identifying a copy number amplification. cfTNA mutations detection, RNA fusion and CNV analysis were performed with the Ion Reporter software using a manufacturer’s provided workflow. (Oncomine TagSeq Lung Liquid Biopsy w2.1—Single Sample, Thermo Fisher Scientific).

### EGFR mutation analysis by cobas

*EGFR* mutation analysis was conducted using the cobas® cfDNA Sample Preparation Kit followed by the cobas® *EGFR* Mutation Test v2 (Roche Molecular Diagnostics), according to the manufacturer’s instructions. The amplification and detection of the mutations was performed in the cobas® 4800 System (Roche Molecular Diagnostics).

### Statistical analysis

For the comparison of mutations detected in paired plasma and tissue biopsy analysis Sensitivity, Specificity, Positive Predictive Value (PPV), and Concordance were calculated. True positives (TP) and true negatives (TN) were defined using the results of the tissue biopsy analysis as a gold standard. False positives (FP) and false negatives (FN) were calculated as the number of mutations detected and not detected respectively by the plasma analysis. Statistics were performed with SPSS (version 20. IBM SPSS STATISTICS). The p-values were based on Fisher’s Exact Test. A p-value <0.05 was considered to be statistically significant.

## Results

### Mutation distribution in plasma of NSCLC patients

**Fig 2:**
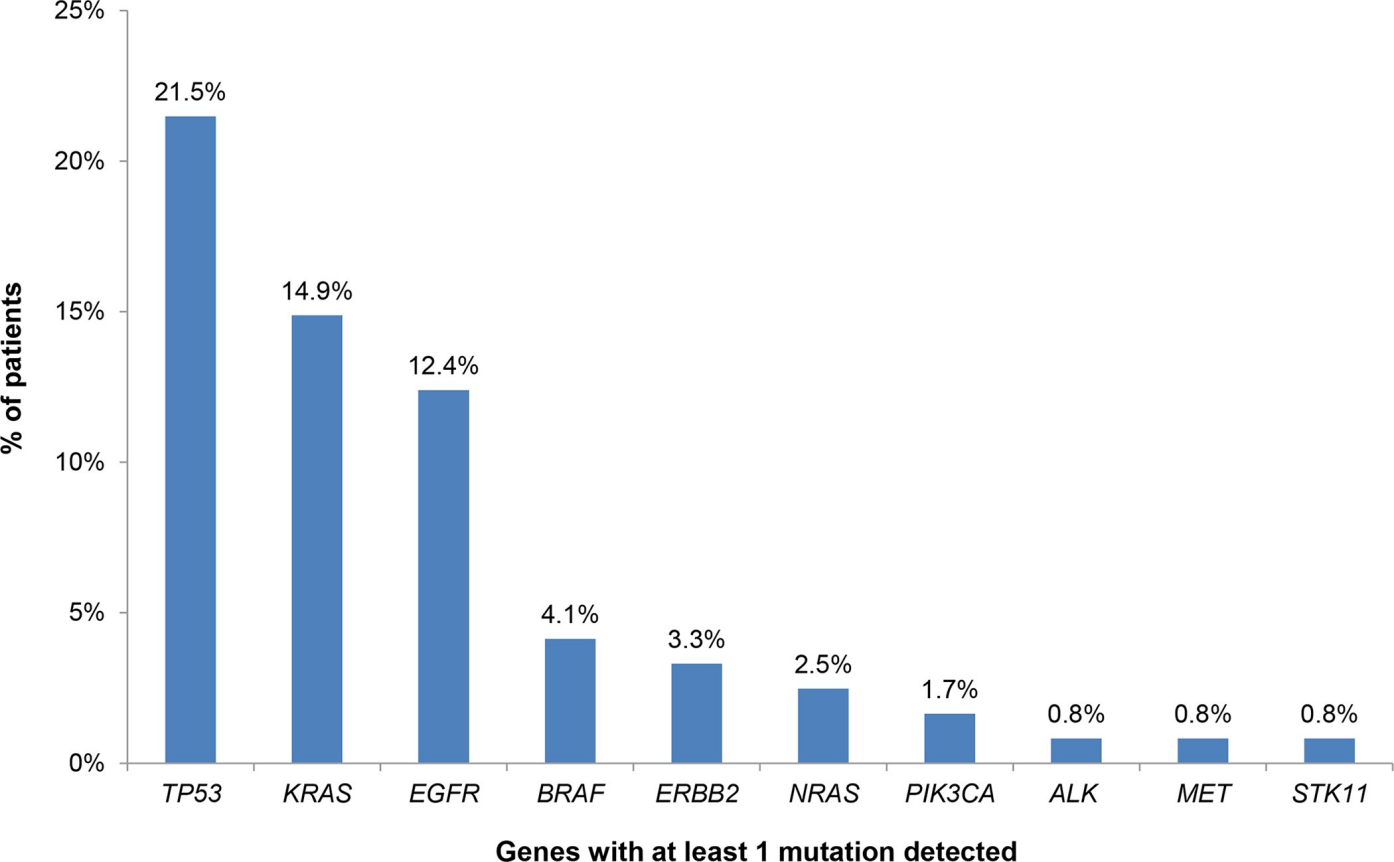
Mutation distribution in plasma samples of 121 NSCLC patients at diagnosis.

Among the 121 patients with newly diagnosed metastatic NSCLC who underwent liquid biopsy analysis, 82 (67.8%) of them were male and 39 (32.2%) were female. The mean age of diagnosis was 67 years. At least one mutation was detected in the plasma of 59 patients (48.76%), of which 74.58% presented only one mutation and 25.42% presented two or more mutations (S1 Table). The percentage of patients with a plasma mutation was similar between males and females (48.78% and 48.72% respectively). *TP53* mutations were the most common alterations (detected in 21.49% of the patients), followed by *KRAS* and *EGFR* gene mutations detected in 14.88% and 12.39% of the cases respectively (Fig 2). The *EGFR* mutation frequency did not vary significantly between males and females (10.76% and 15.38% among males and females respectively).

*EGFR* mutation distribution for exons 18, 19 and 21 was 20,00%, 46.67% and 33.33% respectively. In 2 samples a second mutation in exon 20 (p.S768I) was also present in addition to the sensitizing mutation (exons 18 and 21). *In vitro* and *in vivo* studies have shown that the double mutant receptor is sensitive to treatment with *EGFR* TKIs [30].

The findings obtained by this approach could be used for the assignment of the appropriate treatment based on patients’ molecular profile. The gene alterations analyzed by the NGS methodology used were selected based on their increased prevalence in NSCLC and their possible use as biomarkers of response to targeted treatment. Thus, we have categorized them based on their actionability in 4 categories: those related to on-label treatments, those considered as emerging biomarkers with increasing evidence of predictive value for targeted treatments, those related to clinical trials and those with unknown actionability. Hence, each patient can be assigned to one of these categories on the basis of his mutation profile. In the case of multiple mutations present in the same patient, the categorization is based on the most clinically significant mutation. Using this categorization we observed that 14.88% of the individuals with at least one mutation detected, carried a mutation related to an approved treatment for NSCLC. These include sensitizing mutations in the *EGFR* gene, the p.V600E mutation in the *BRAF* gene and ALK fusions (in 12.39%, 1.65% and 0.83% of the patients respectively). In 3.31% of the patients tested, a mutation in the *ERBB2* gene was detected. There is increasing evidence for the utility of the alterations in this gene as a biomarker of response to targeted treatment and thus it is reported as an emerging biomarker for NSCLC in the NCCN guidelines (www.nccn.org). Furthermore, in 37 patients (30.58%) the mutations detected could be related to off-label treatment or a clinical trial. More precisely, these mutations were located in the *TP53*, *KRAS*, *NRAS*, *BRAF* (non-V600E), *STK11* and *PIK3CA* genes.

### Concordance in mutation distribution between tissue and plasma

**Fig 3:**
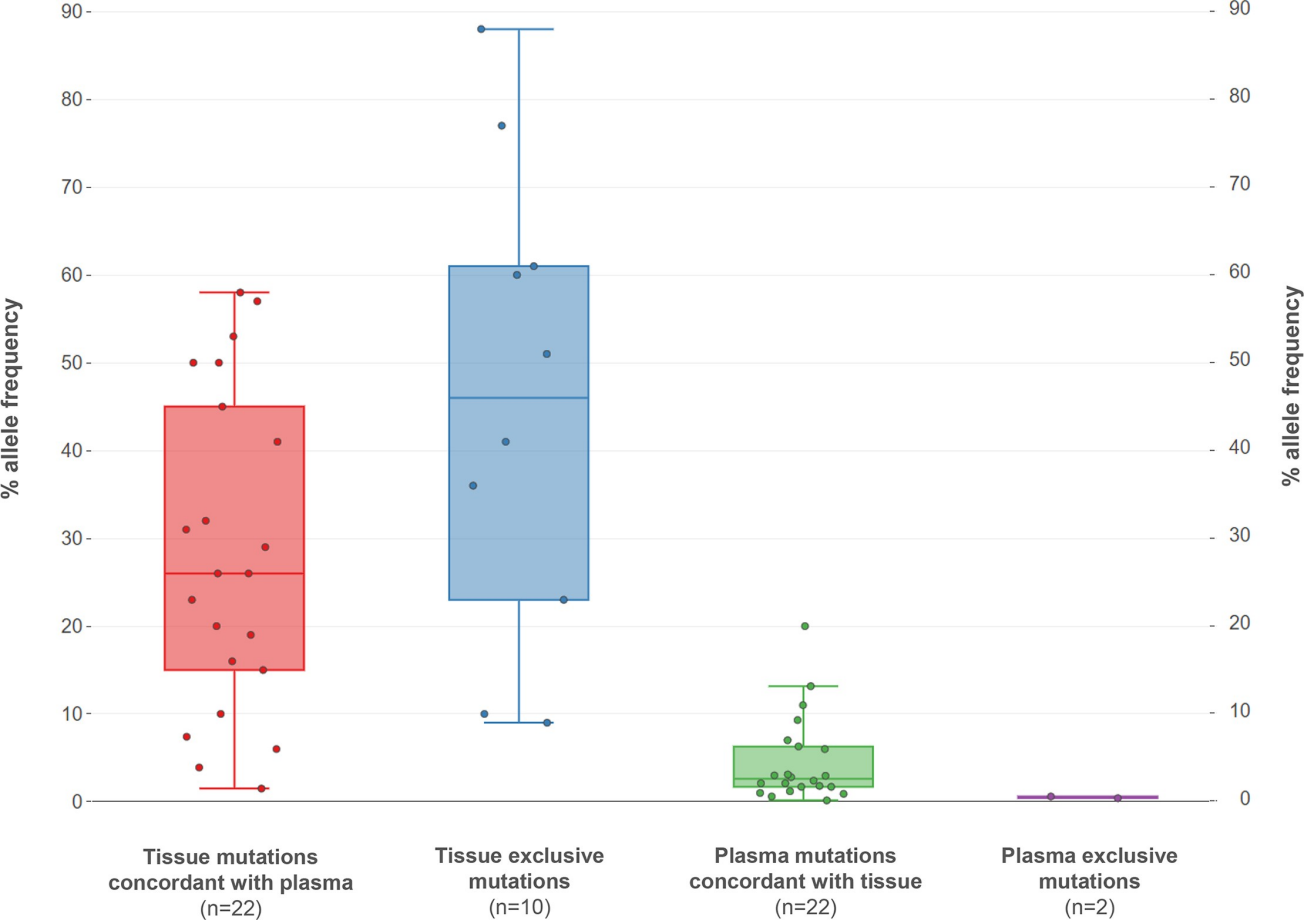
Median allele frequency of mutations detected in tissue and plasma grouped based on their concordance among the two materials.

In the 36 patients with concomitant tissue and liquid biopsy available, a total of 34 mutations were detected. Of them, 22 mutations were detected in both materials, 10 mutations were detected in tissue only and 2 mutations were detected in plasma only (Fig 3). The mean mutated allele fraction was 33.62% for somatic mutations detected in tumor biopsy, while this value, as expected, was significantly lower in plasma samples (4.22%). However, the detection of a mutation in plasma did not correlate with its percentage in tissue as there was not a statistically significant difference in the % allele frequency between tissue exclusive mutations and concordant with plasma tissue mutations (p = 0.2695) (Fig 3).

**Fig 4:**
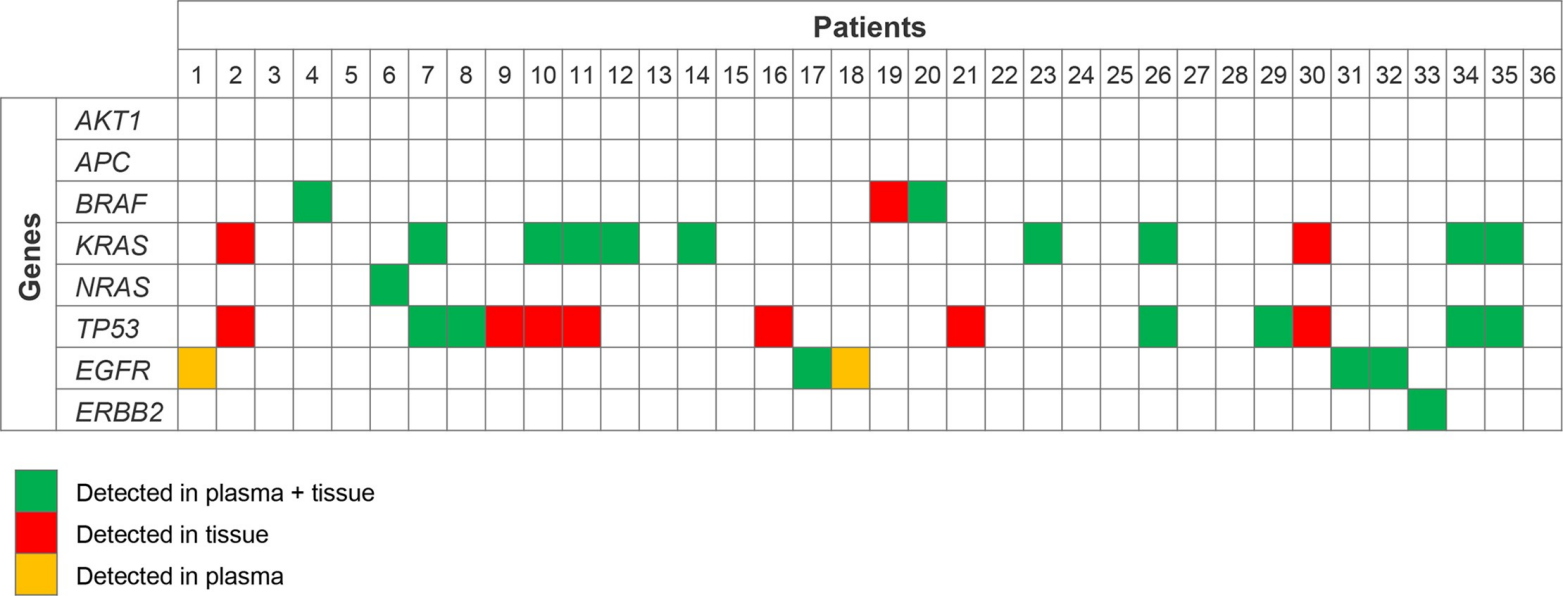
Comparison of the mutation distribution in plasma and tissue of 36 patients with newly diagnosed NSCLC.

The concordance between these 2 materials varied depending on the gene analyzed. The lowest concordance rate in the mutational status between tissue and plasma was observed for the *TP53* gene (67.44%), where 7 out of the 13 mutations detected in tissue were not present in the plasma (sensitivity 46.15%, specificity 100%) (Table 1, Fig 4). On the contrary, the concordance rate between tissue-plasma for the *KRAS* and *NRAS* status was 94.74%, with an 85.71% sensitivity of *RAS* mutation detection in plasma in case of a positive tissue result and 100% specificity. Notably, all 5 *EGFR* mutations detected in the tissue were also present in plasma (sensitivity 100%); however, in 2 cases, an *EGFR* mutation was detected in plasma while it was not present in the tissue sample. Considering the tissue as the gold standard material, we could assign a specificity rate for these mutations of 93.94%.

**Table 1:** Sensitivity, specificity, PPV (Positive Predictive Value) and concordance for mutations detected in paired plasma and tissue biopsy analysis.

**Gene(s)**	Sensitivity	Specificity	PPV	Concordance
**KRAS/NRAS**	85.71%	100%	100%	94.74%
**EGFR**	100%	93.94%	60%	94.44%
**TP53**	46.15%	100%	46.15%	67.44%
**Clinically Significant Genes**	84.21%	88.24%	88.89%	86.11%
**All Genes**	68.75%	83.33%	91.67	72.73%

In summary in both tissue and plasma, the detection of clinically significant mutations was possible. Considering only genes with approved or emerging targeted treatments available, such as *EGFR*, *BRAF* and *ERBB2* and genes with prognostic relevance such as *KRAS* and *NRAS* genes, we can observe a concordance rate among tissue and plasma for their mutational status of 86.11% with a Positive Predictive Value (PPV) of 88.89%.

### Concordance of plasma mutation distribution between cobas and NGS approaches

Another point of investigation of this study was the feasibility of liquid biopsy analysis in patients that harbored an *EGFR* mutation at diagnosis and have relapsed following treatment with *EGFR* TKIs. In these cases, a very common clinical practice is the use of the CE-IVD cobas^®^ *EGFR* Mutation Test, in order to detect the T790M resistance mutation in the *EGFR* gene. Thus, we compared the results obtained by cobas and by our NGS approach in patients that were referred to our laboratory, following treatment with *EGFR* TKIs, due to suspected insensitivity to the given treatment. Of note, in the majority of the cases, the initial *EGFR* mutation analysis was performed in a different laboratory, thus we were not aware of the primary sensitizing *EGFR* mutation nor in position to verify it.

**Fig 5:**
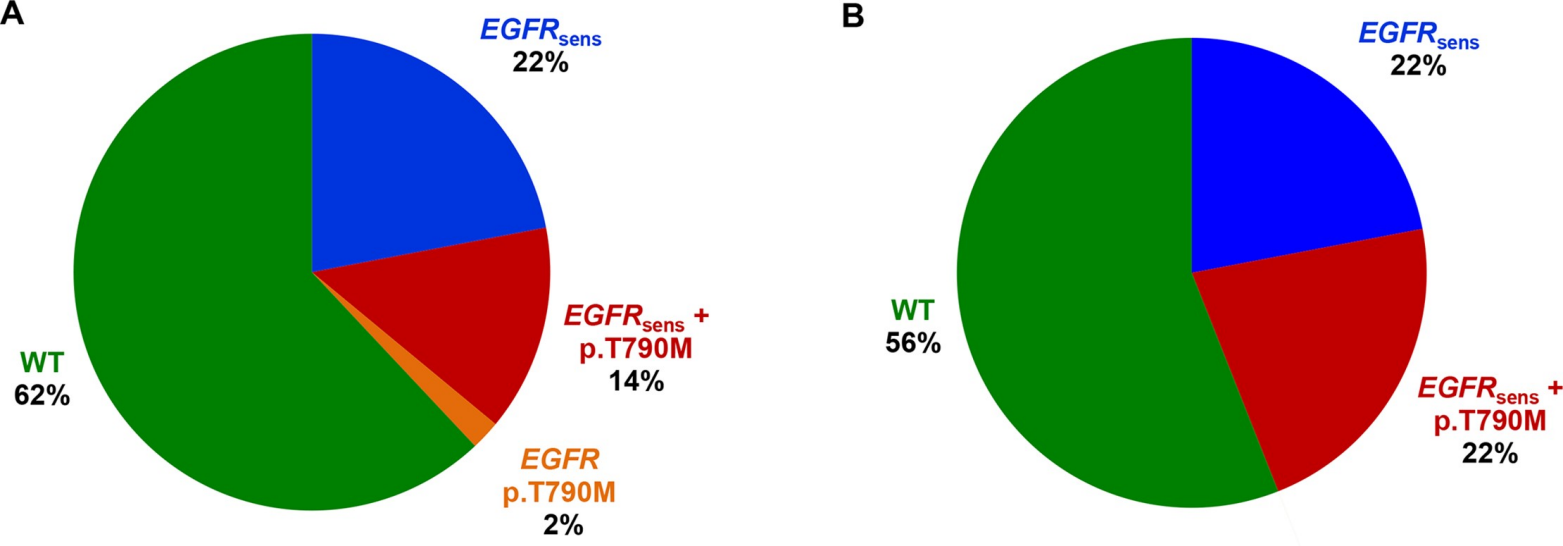
EGFR mutation distribution in ctDNA of 50 patients with an EGFR mutation at diagnosis that have relapsed following TKI treatment. A. Analysis by cobas B. Analysis by NGS.

When the *EGFR* analysis by cobas was applied in this patients’ group, an *EGFR* mutation was detected in 38% of the cases, while the primary sensitizing *EGFR* mutation was absent in 62% of the cases. Among patients with an *EGFR* mutation detected, 11 harbored a sensitizing *EGFR* mutation, 7 harbored an *EGFR* sensitizing mutation plus the T790M resistance mutation, and 1 harbored the T790M resistance mutation alone (Table 2, Fig 5).

**Table 2:** Gene mutation results obtained by cobas and NGS methods in 50 consecutive NSCLC patients referred for T790M resistance mutation analysis due to relapse after TKI treatment.

PATIENT	EGFR analysis by cobas^a^	EGFR analysis by NGS^b^	Other Gene Alterations detected by NGS^c^
1	Ex19Del (12.82)	Ex19Del (p.L747_S752del) [9.62%]	*BRAF* p.V600K [0.6%]
2	p.L858R (6.00)	Wild Type	*KRAS* p.G12V [0.5%]
3	p.S768l (5.49), p.G719X (2.54)	p.G719S [5.23%]	NONE
4	p.L858R (6.97)	p.L858R[6.72%]	NONE
5	p.L861Q (5.21)	p.L861Q [0.38%]	NONE
6	Ex19Del (19.01)	Ex19Del (p.Glu746_Ala750del) [27.71%]	*TP53* p.Y220C [0.9%]
7	Ex19Del (17.49)	Ex19Del p.Glu746_Ala750del [23.44%]	NONE
8	Ex19Del (5.99)	Ex19Del p.E746_A750del [9.12%]	*TP53* p.R175H [1.1%]
9	Ex19Del (5.99)	Wild Type	NONE
10	Ex19Del (10.12)	Ex19Del (p.E746_A750del) [20.57%], p.T790M [0.55%]	NONE
11	EX19Del (10.96), p.T790M (15.28)	Ex19Del (p.L747_A750delinsP) (24.42%), p.T790M (12.78%)	NONE
12	EX19Del (6.00), p.T790M (8.33)	Ex19Del(p.L747_T751del) (0.82%), p.T790M (1.24%)	NONE
13	p.L858R (13.02), p.T790M (16.84)	p.L858R (2.44%), p.T790M (4.55%)	NONE
14	EX19Del (12.04), p.T790M (17.02)	Ex19Del (p.E746_A750del) (11.55%), p.T790M (10.66%)	NONE
15	EX19Del (14.56), p.T790M (12.73)	p.L858R (4.45%), p.T790M (3.12%)	NONE
16	EX19Del, p.T790M (11.2)	Ex19Del (p.L747_T751>Q) (11.52%), p.T790M (2.45%)	NONE
17	EX19Del (11.36), p.T790M (16.30)	Ex19Del (p.E746_A750del) (2.98%), p.T790M (3.21%)	NONE
18	EGFR p.T790M (5.66)	Ex19Del (p.E746_A750del) (1.04%), p.T790M (1.56%)	NONE
19	Wild Type	p.L858R [9.63%], p.T790M [1.24%]	NONE
20	Wild Type	Wild Type	*BRAF* p.V600E [0.8%]
21	Wild Type	Wild Type	NONE
22	Wild Type	Wild Type	NONE
23	Wild Type	Wild Type	*TP53* p.C176S [1.9%]
24	Wild Type	Wild Type	NONE
25	Wild Type	Wild Type	*MET* amplification [1.3]
26	Wild Type	Wild Type	NONE
27	Wild Type	Wild Type	*KRAS* p.G12D [0.7%]
28	Wild Type	Wild Type	NONE
29	Wild Type	Ex19Del (p.E746_A750del) [9.24%], p.T790M [1.65%]	NONE
30	Wild Type	Wild Type	NONE
31	Wild Type	Wild Type	NONE
32	Wild Type	Wild Type	NONE
33	Wild Type	Wild Type	NONE
34	Wild Type	Wild Type	*MET* AMPLIFICATION [1.64]
35	Wild Type	Wild Type	NONE
36	Wild Type	Wild Type	NONE
37	Wild Type	Wild Type	*NRAS* p.G13S [0.82%]
38	Wild Type	Ex19Del (p.E746_S752delinsV) [0.72%]	*TP53* p.G245D [0.83%], *MET* p.Y1248H [0.94%], *KRAS* p.G12C [0.63%], *PIK3CA* p.E542K [1.02%]
39	Wild Type	Wild Type	NONE
40	Wild Type	Wild Type	*TP53* p.R249fs [3.82%]
41	Wild Type	Wild Type	NONE
42	Wild Type	Wild Type	NONE
43	Wild Type	Wild Type	*TP53* p.R282W [2.54%]
44	Wild Type	Wild Type	NONE
45	Wild Type	p.G719A [4.12%]	*KRAS* p.G12V [2.33%], *BRAF* p.V600E [1.64%], *TP53* p.R175H [1.23%]
46	Wild Type	Ex19Del (p.E746_S750del) [0.47%]	MAP2K1 p.E203K [0.82%]
47	Wild Type	Wild Type	NONE
48	Wild Type	Wild Type	NONE
49	Wild Type	p.G719A [0.93%]	*PIK3CA* p.E542G [0.44%], *TP53* p.R175H [0.85%], *KRAS* p.G12C [0.66%]
50	Wild Type	Wild Type	NONE

a. The semi-quantitative index of the cobas test is provided in brackets.

b. Allele frequency of EGFR gene detected by the NGS method is given in brackets.

c. Allele frequency of each gene alteration and/or the CNV ratio of each amplification detected is given in brackets.

*EGFR* analysis by NGS in the same cohort revealed the presence of an *EGFR* mutation in 44% (22/50) of the cases. In the mutation positive group, 50% of the patients carried a sensitizing mutation while a sensitizing plus a resistance mutation was present in the rest of the cases.

The concordance rate between the two methods in the *EGFR* mutation analysis was 82% for the *EGFR* sensitizing mutations (in exons 18, 19, 21). In some cases the discordances concerned low frequency variants with an allele frequency <1% (as measured by NGS) or with a cobas semi-quantitative index < 6. In 2 cases an *EGFR* sensitizing mutation was not detected by NGS while it was detected by cobas. Furthermore, in 6 cases the sensitizing mutation was only detectable by the NGS methodology.

For the p.T790M mutation, a higher concordance between the two approaches was observed (94%), with the NGS analysis being able to detect the p.T790M mutation in 3 additional patients.

**Fig 6:**
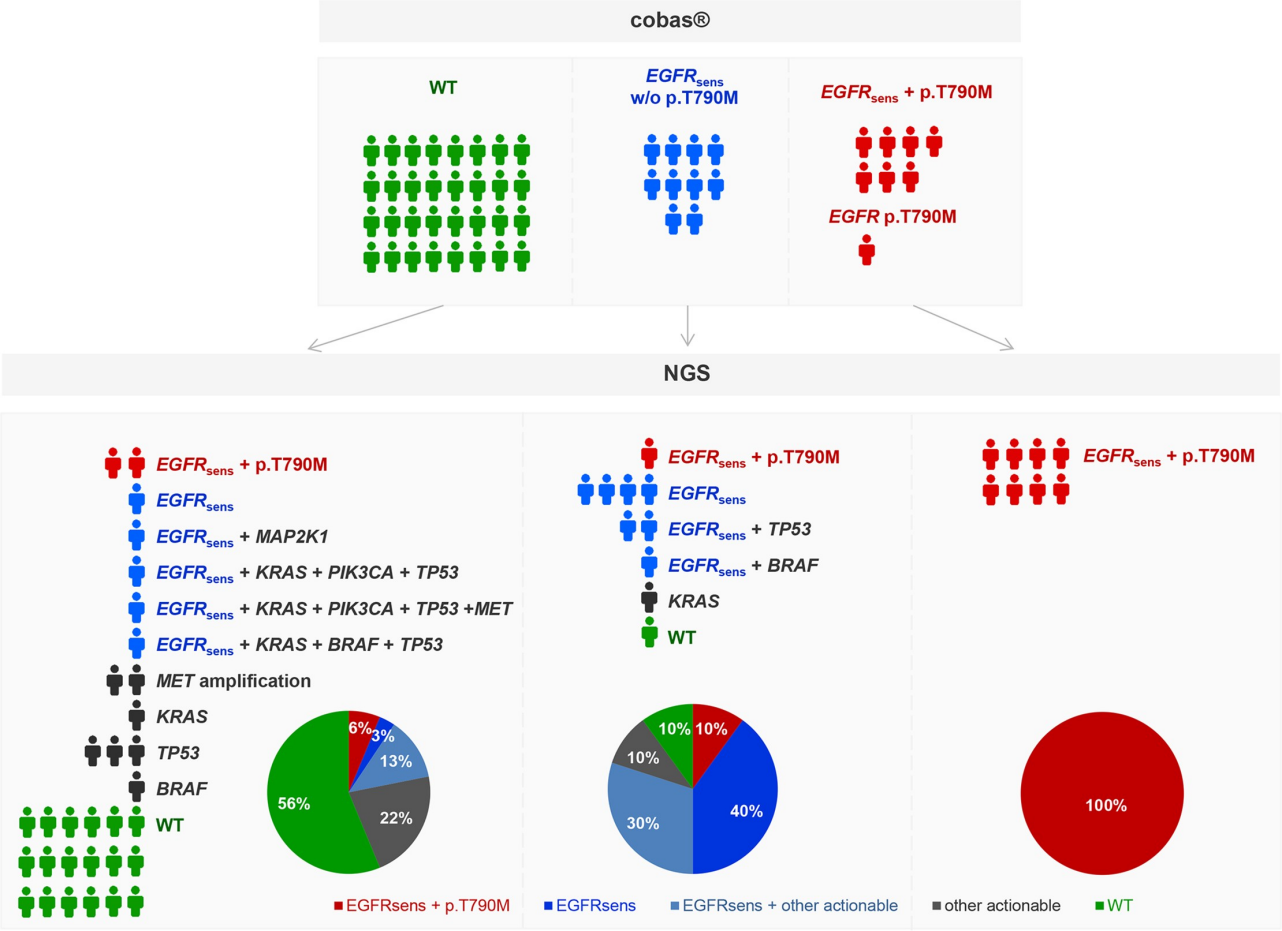
NGS-cobas comparison in 50 EGFR mutant NSCLC patients in relapse following EGFR TKIs treatment. EGFR sens.: EGFR sensitizing mutations in exons 18, 19, 21.

In contrast to the cobas assay, NGS analysis is a multigene assay that is also providing information for other important and possibly clinically relevant cancer genes. Such approach allows the detection of additional mutations besides the T790M mutation that could contribute to resistance to EGFR TKI treatment. This is of significant importance in cases without the T790M mutation detected. In our cohort, in the 39 patients without the T790M mutation (10 with only an *EGFR* sensitizing mutation present and 32 without any mutation detected), mutations in the *KRAS* and *BRAF*, *PIK3CA*, *MAP2K1* genes as well as a *MET* amplification (Table 2) where identified. These alterations could explain the unresponsiveness to treatment in these patients, thus clarifying the resistance mechanism. Additionally, some of these alterations such as *BRAF* and *MET* amplification are targetable and could be used for the enrollment of these patients to clinical trials (Fig 6).

## Discussion

Several previous studies have shown the clinical utility of liquid biopsy analysis using multigene approaches [31–33]. However, its predictive value for genes other than *EGFR* is still not widely recognized [34, 35]. In the current study, we report a single center’s experience of using this type of analysis for the detection of clinically significant alterations in NSCLC patients. Hence, plasma analysis of 171 NSCLC patients was performed using a sensitive NGS assay that analyses hotspot regions of 12 genes frequently altered in NSCLC and fusions in *ALK*, *ROS1* and *RET* genes.

The NGS method applied in this study employs random molecular tags that are used as unique molecular identifiers (UMI) to uniquely label each molecule prior to library amplification. In this way, randomly incorporated errors can be distinguished (and removed) from true variants, increasing the method’s accuracy and minimizing false positives [34, 36, 37]. The variant detection limit of the assay is 0.1%, with 90% sensitivity and >98% specificity for Single nucleotide variation hotspots and small insertions/deletions [27, 38]. Nevertheless, cautious selection of the appropriate NGS method is indispensable for obtaining accurate molecular analysis results using plasma as a starting material. Not all methods have the same sensitivity and specificity and traditional amplicon based NGS techniques without the addition of unique molecular identifiers seem to be less appropriate for use in liquid biopsy analysis [39]

Multi-gene analysis can be achieved in less than a week, allowing physicians to make quick treatment decisions. Additionally, the clinical information provided is superior compared to that obtained from the analysis of only one gene. The price of such analysis depends mainly on the number of genes analyzed and the methodology used. In any case, the cost per gene is much lower than the single-gene analysis. Therefore, we believe that plasma NGS methods analyzing a small group of targetable genes specific for each tumor type are cost-effective. Thus, their application in clinical practice is feasible.

**Fig 7:**
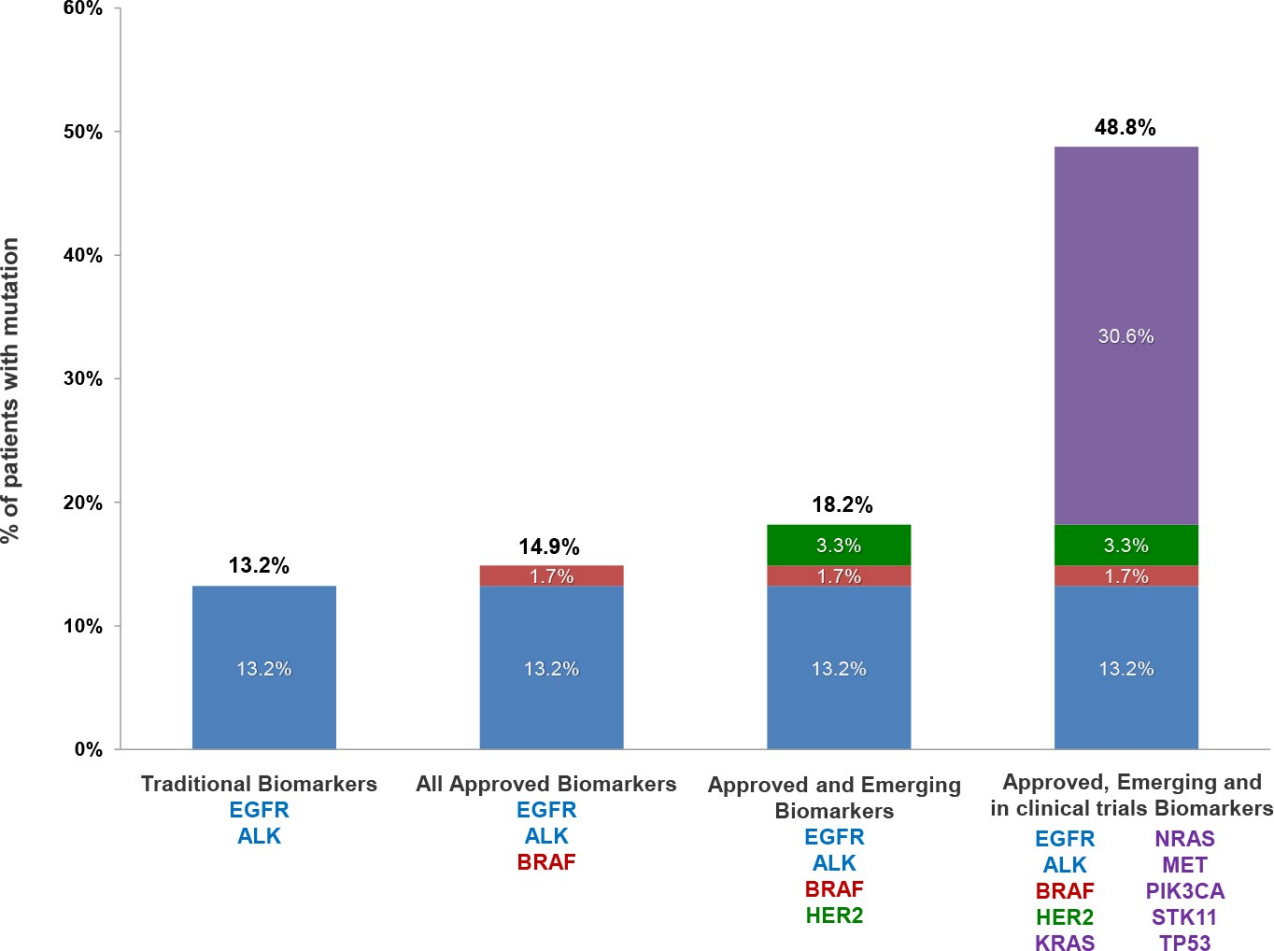
Apportionment of the 59 NSCLC patients with at least one variant identified in liquid biopsy analysis, using 4 different biomarker categories: traditional targeted treatment biomarkers (*EGFR*, *ALK*); all approved treatment associated biomarkers (*EGFR*, *ALK*, *BRAF*); approved and emerging treatment biomarkers (*EGFR*, *ALK*, *BRAF*, *HER2*); approved, emerging treatment & clinical trials associated biomarkers (*EGFR*, *ALK*, *BRAF*, *HER2*, *KRAS*, *NRAS*, *MET*, *PIK3CA*, *STK11*,*TP53*). In case a patient harbors more than one mutation the categorization is based on the more clinically significant variant.

The simultaneous analysis of many therapeutic targets by this methodology in NSCLC, a tumor type with various treatment options available, is of great clinical significance. NGS technology rendered this analysis possible even in a material such as plasma with minute concentrations of genetic material available. However, physicians often require the examination only of traditional biomarkers such as *EGFR* and *ALK*. Nevertheless, if the analysis was limited to these genes, a mutation would have been detected in only 13.2% of the cases (Fig 7). Furthermore, a recently approved biomarker for NSCLC is the p.V600E mutation in the *BRAF* gene. The analysis of this variant increases the percentage of patients positive for an approved biomarker to 14.89% (Fig 7). *ERBB2* mutations in NSCLC, mainly consist of exon 20 in frame insertions and are present in 1–3% of the patients [40]. Additionally *MET* exon14 skipping mutations and amplification are also present in 1–4% of the cases [41–43]. Both *ERBB2* and *MET* alterations are considered emerging biomarkers of responsiveness to treatment and are included in the NCCN guidelines (www.nccn.org). In our cohort *ERBB2* and *MET* alterations were present in 3.31% and 0.83% of the cases respectively (Fig 7).

Further biomarker analysis could also contribute to the clarification of the patient’s genetic profile and give additional clinical important information. For example the analysis of *KRAS* mutations, which is present in 15–25% of the patients, is suspected to be a prognostic biomarker of worse treatment outcome in NSCLC adenocarcinoma [44]. Additionally, there are studies showing that patients harboring this mutation are unlike to respond to targeted treatment with *EGFR* TKIs [45, 46]. Clinical trials also exist for other biomarkers in our cohort (www.clinicaltrials.gov). The BFAST phase II/III clinical trial is an example of such an effort, aiming to investigate the clinical utility of actionable alterations’ identification using plasma NGS analysis without the influence of tissue-based testing (NCT03178552). NSCLC patients with an actionable alteration detected in plasma are assigned to the matched targeted treatment or immunotherapy and treatment’s benefit is calculated. Recently, the first results announced from this study in the ALK-positive cohort indicated that liquid biopsy analysis can efficiently detect such alteration and the plasma positivity in *ALK* rearrangements is highly predictive of alectinib response.

Several studies have confirmed the clinical utility of ctNA analysis for the detection of tumor originated mutations in NSCLC as well as in other tumor types [3, 19, 47–50]. In accordance with these results, in the 36 patients with concurrent tissue and plasma available we observed a 72.73% concordance rate among the two materials in the mutations detected. This concordance is even higher when considering the clinically relevant mutations with an approved or investigational targeted treatment available or with prognostic value (*EGFR*, *BRAF*, *ERBB2*, *KRAS*, *NRAS*). The higher concordance rate could be attributed to the fact that these are driver mutations that occur early in cancer development [51]. A sensitivity of 84.2% was achieved for these mutations with a concordance rate of 88.9%. This is of particular importance since these mutations will presumably be used for treatment decision making. Even though, the tissue is considered as the gold standard material for specificity calculation in tissue versus liquid biopsy concordance studies, this assumption does not take into account tumor heterogeneity, that could result in omission of mutations when using tissue biopsies [52]. This is even more prominent for NSCLC, which presents several tissue sampling issues since the majority of the cases are inoperable and the analysis is often performed using limited size biopsies with less than 100–150 tumor cells present [53]. This was also the case of our two negative *EGFR* tissue results with discordant plasma outcomes. Unfortunately, the physicians relied on the tissue result, since it was considered more trustworthy for treatment decision, rather on the liquid biopsy result. In the same way, no targeted treatment was administrated in the 3 patients with *ERBB2* mutations detected (2 in liquid biopsy only and one in both tissue and plasma), even though these variations are considered emerging biomarkers of response to ado trastuzumab emtansine [54] (www.nccn.org/). This was attributed by the physicians to the absence of approval of this regiment for NSCLC and to the costly and/or lengthy procedures required for administrating the drug off-label or for the enrollment in a clinical trial.

In a recent study though, it has been shown that plasma driven targeted treatment administration in NSCLC patients showed effectiveness in 36 of the 42 patients with evaluable results (85.7%), including stable disease partial and complete response in 16, 19 and 1 case respectively. Furthermore, the depth of RECIST response was not correlated to the mutation allele frequency in plasma 49]. Similarly, Reckamp and colleagues showed that patients’ response to third generation EGFR TKI is achieved, irrespectively of whether the T790M mutation is detected in tissue, plasma or urine samples [55]. Hopefully, the issue of liquid biopsy results’ credibility will be resolved soon, since currently there are more than 130 ongoing clinical trials that are exploring the role of such analysis in different tumor types and probably upon completion will highlight its importance, especially in cases with no tissue available (www.clinicaltrial.gov). Recently, results from the SOLAR-1 phase III randomized controlled trial (NCT02437318) indicated a significant improvement in Progression Free Survival (PFS) from the addition of alpelisib to the fulvestrant treatment in HER2 negative, Hormonal Receptors (HR) positive Breast Cancers patients who harbored a *PIK3CA* mutation accessed either using tissue or plasma. Thus, *PIK3CA* became the second targetable gene (after *EGFR*) with an approved drug available using liquid biopsy analysis for the detection of the alteration targeted [56]. The accumulation of such data enhances the position of the non-invasive tumor molecular profiling approach and will eventually lead to more drug approvals based on liquid biopsy biomarkers.

The most recognized application of ctDNA analysis is in the detection of mutations that arise due to the *EGFR* TKIs targeted treatment, with the p.T790M mutation being the best studied resistant mechanism, since it provides sensitivity to third-generation *EGFR* TKIs [57]. Osimertinib, was the first third-generation EGFR TKI, to be approved for patients that harbor the p.T790M mutation and have progressed following EGFR TKIs treatment. Recently it has also been approved as first-line therapy in EGFR mutated NSCLC patients, irrespectively of the T790M mutation status [58, 59]. Nonetheless, several issues concerning this TKI remain unclear such as the resistance mechanism of this treatment when received in first line and the consequences of its prolonged administration. The ongoing MELROSE: Phase 2 clinical trial aims to clarify the resistance mechanisms of these medications, analyzing both tissue and plasma ctDNA (NCT03865511). The first and only FDA approved kit for *EGFR* mutation analysis is the cobas^®^ *EGFR* Mutation Test v2 from Roche Diagnostics, which is a real-time PCR assay designed to detect 42 *EGFR* mutations in exons 18, 19, 20 and 21, including the resistant T790M mutation. This assay has shown good performance in *EGFR* mutation analysis across different studies [20, 22–24]. However, it should be noted that the sensitivity of this assay does not exceed 77% for the *EGFR* exon 19 and 21 mutations, with the sensitivity being much lower, around 61% for the pT790M resistance mutation (20). As a result, if a p.T790M mutation is present in plasma there is almost 40% of probability to miss it, by the cobas analysis. An additional cause of missing tissue mutations in plasma is the phenomenon of Non-Shedding tumors, that release only limited amount of ctDNA in circulation [60]. Thus, current guidelines recommend a re-biopsy in case of negative plasma result when accessing resistance in NSCLC patients at relapse upon EGFR TKI treatment (www.nccn.org) [61]. In order to reduce the incidence of invasive re-biopsies in these cases the use of more sensitive techniques should be applied with NGS providing an excellent alternative to the cobas method. In the recent years several modified NGS approaches with increased sensitivity and specificity have been applied for ctDNA analysis [27, 28, 62], showing a better accuracy on the plasma for the T790M mutation identification [63]. In addition to the increased sensitivity, the NGS methodology offers the advantage of being a multigene assay, providing more clinically relevant information compared to a single gene test. It is known that p.T790M mutation is only one of the existing resistant mechanisms developed by the tumor following to EGFR TKI treatment. In 45–50% of the cases, the resistance arises through different mechanisms and can be due to *EGFR*-independent mechanisms, including alterations in genes involved in alternative pathways (such as *PIK3CA*, *BRAF*, *KRAS*, *ERBB2*, and *MET*) and histological or phenotypic transformation [11, 64].

In order to evaluate the concordance rate among these two platforms, 50 NSCLC patients that were positive for an *EGFR* sensitizing mutation at diagnosis and had stopped responding to *EGFR* TKI treatment were analyzed by both cobas and NGS platforms. An 82% concordance was observed in *EGFR* sensitizing mutations identification (exons 18, 19, 21) between the two assays, in plasma. The concordance for the p.T790M mutation was higher reaching a percentage of 94%. In 6 cases the sensitizing mutation was detected only by NGS analysis, whereas only 2 sensitizing EGFR mutations were missed by this approach. Most importantly, 3 additional p.T790M mutations were identifiable only by the NGS methodology. In these cases a third-generation *EGFR* TKI could be used to overcome resistance to TKI treatment, revealing the importance of using more sensitive approaches for plasma analysis. In one case, an eight-month response to osimertinib was achieved, and in a subsequent liquid biopsy analysis recommended by the physician due to loss of responsiveness, the C797S mutation was detected, which is responsible for resistance to third-generation *EGFR* TKIs. This mutation would have been missed if the analysis was carried out by cobas since it is not included in the mutations analyzed by this assay.

Moreover, NGS analysis of the 42 patients without the p.T790M resistance mutation, revealed mutations in other genes. Those alterations were located in *TP53*, *KRAS*, *BRAF*, *MET*, *PIK3CA*, *NRAS*, and *MAP2K1* genes, in 19.05%, 11.90%, 7.14%, 7.14%, 4.76%, 2.38% and 2.38% of the cases respectively. The majority of these alterations have been reported previously as resistance mechanisms to *EGFR* treatment [11, 64]. Therefore, the resistance mechanism could be elucidated in more patients by the use of a multigene assay. Additionally, among the alterations detected in this cohort, the *BRAF* p.V600 mutations and the *MET* amplifications are candidate predictive markers of response to *BRAF* and *MET* inhibitors respectively in this patients’ setting [41, 65, 66]. Thus, the addition of such inhibitors could be considered to overcome *EGFR* TKI resistance in these patients [67, 68].

In conclusion, this study provides evidence of the feasibility of ctNA analysis as a tumor biopsy surrogate in clinical practice for NSCLC personalized treatment decision making. NGS is a technology that can provide a comprehensive molecular characterization of the tumor using both tissue and plasma. The applicability of this approach in clinical practice is shown by the significant percentage of patients with a targetable mutation detected both before and after targeted treatment. Thus, it could aid physicians in treatment decision making in clinical practice.

## Supporting information

S1 Table Gene alterations detected by NGS in plasma samples of 121 NSCLC patients. (XLSX)
